# How Low Should We Go to Prevent Brain Aging Associated with Exposure to Ambient Fine Particles?

**DOI:** 10.1002/alz.095433

**Published:** 2025-01-09

**Authors:** Xinhui Wang, Jiu‐Chiuan Chen, Lauren Salminen, Andrew J Petkus, Joshua Millstein, Mark A. Espeland, Meredith N Braskie, Paul M. Thompson, Margaret Gatz, Susan M. Resnick, Joel D. Kaufman, Stephen R. Rapp, Janet A Tooze, Sally A. Shumaker, Diana Younan

**Affiliations:** ^1^ USC Keck School of Medicine, Los Angeles, CA USA; ^2^ University of Southern California, Los Angeles, CA USA; ^3^ Imaging Genetics Center, Mark and Mary Stevens Neuroimaging and Informatics Institute, Keck School of Medicine, University of Southern California, Marina del Rey, CA USA; ^4^ Wake Forest University School of Medicine, Winston Salem, NC USA; ^5^ Stevens Neuroimaging and Informatics Institute, Los Angeles, CA USA; ^6^ Imaging Genetics Center, Mark and Mary Stevens Neuroimaging & Informatics Institute, University of Southern California, Marina del Rey, CA USA; ^7^ Laboratory of Behavioral Neuroscience, National Institute on Aging, Intramural Research Program, Baltimore, MD USA; ^8^ University of Washington, Seattle, WA USA; ^9^ Wake Forest University School of Medicine, Winston‐Salem, NC USA; ^10^ Wake Forest School of Medicine, Winston‐Salem, NC USA; ^11^ Amgen Biotechnology Research, Thousand Oaks, CA USA

## Abstract

**Background:**

Late‐life exposure to PM_2.5_ is a risk factor for Alzheimer’s disease (AD) and related dementias. Accelerated brain aging associated with PM_2.5_ exposure likely takes place at preclinical stage. In Feb.2024, US EPA lowered the annual standard for fine particulate matter (PM_2.5_) from 12‐ to 9‐µg/m^3^. Whether the new exposure standard is sufficient to protect the aging brain is unclear.

**Method:**

This current study examined whether PM_2.5_‐associated brain aging is present at lower‐level exposures. We used longitudinal data from older women (n = 421; aged 77.3±3.5) enrolled in the Women’s Health Initiative Memory Study MRI‐1 (2005‐6) and MRI‐2 (2009‐10), living at locations with PM_2.5_ below 12‐µg/m^3^ (including 130 complying with EPA’s new standard) and without MCI, dementia or stroke prior to MRI‐1. PM_2.5_ exposures at residential locations were estimated as 3‐year annual averages prior to MRI‐1, based on validated regionalized universal kriging models. AD pattern similarity (AD‐PS) scores, developed by supervised machine learning and validated with ADNI data, were used to assess AD‐related neurodegeneration. Bilateral gray matter volumes of the hippocampus, amygdala, parahippocampal gyrus (PHG), and entorhinal cortex were summed to define the regional volume of medial temporal lobe (MTL). We used linear regressions to estimate exposure effects on 5‐year changes in the AD‐PS scores and atrophies of MTL and its subregions, adjusting for intracranial volume, sociodemographic, lifestyle, and clinical characteristics.

**Result:**

During 5 years of follow‐up, on average AD‐PS scores increased by 0.16±0.15, while MTL volume shrank by 0.42±1.02 cm^3^. Among older women with PM_2.5_ <12‐µg/m^3^, each 1‐µg/m^3^ increment was associated with increased AD‐PS scores (β_AD‐PS_ = 0.013, 95%CI = [0.003, 0.021], p<0.01) and additional MTL shrinkage (β_MTL_ = ‐0.079, 95%CI = [‐0.138, ‐0.019], p = 0.01; primarily affecting PHG), equivalent to 3.0%‐3.6% higher dementia risks. PM_2.5_ exposure < 9‐µg/m^3^ remained significantly associated with progressive PHG atrophy (β_PHG_ = ‐0.087, 95%CI = [‐0.169, ‐0.005], p = 0.04), but the associations with increased AD‐PS score or progressive MTL atrophy had less precise effect estimates. Further restricting the analyses to older women who remained cognitively normal at MRI‐2 did not materially change the results.

**Conclusion:**

Our study supports EPA’s new decision to lower PM_2.5_ standard, but could not rule out the possibility of neurotoxic effects on aging brain with PM_2.5_ <9‐µg/m^3^.

